# Systematic investigation of insertional and deletional RNA-DNA differences in the human transcriptome

**DOI:** 10.1186/1471-2164-13-616

**Published:** 2012-11-13

**Authors:** Cai Chen, Ralf Bundschuh

**Affiliations:** 1 Biophysics Graduate Program, The Ohio State University, Columbus, OH, USA; 2 Departments of Physics and Chemistry & Biochemistry, The Ohio State University, Columbus, OH, USA; 3 Center for RNA Biology, The Ohio State University, Columbus, OH, USA

**Keywords:** Indel RNA-DNA differences, RNA-seq data analysis, Computational filtering

## Abstract

**Background:**

The genomic information which is transcribed into the primary RNA can be altered by RNA editing at the transcriptional or post-transcriptional level, which provides an effective way to create transcript diversity in an organism. Altering can occur through substitutional RNA editing or via the insertion or deletion of nucleotides relative to the original template. Taking advantage of recent high throughput sequencing technology combined with bioinformatics tools, several groups have recently studied the genome-wide substitutional RNA editing profiles in human. However, while insertional/deletional (indel) RNA editing is well known in several lower species, only very scarce evidence supports the existence of insertional editing events in higher organisms such as human, and no previous work has specifically focused on indel differences between RNA and their matching DNA in human. Here, we provide the first study to examine the possibility of genome-wide indel RNA-DNA differences in one human individual, NA12878, whose RNA and matching genome have been deeply sequenced.

**Results:**

We apply different computational tools that are capable of identifying indel differences between RNA reads and the matching reference genome and we initially find hundreds of such indel candidates. However, with careful further analysis and filtering, we conclude that all candidates are false-positives created by splice junctions, paralog sequences, diploid alleles, and known genomic indel variations.

**Conclusions:**

Overall, our study suggests that indel RNA editing events are unlikely to exist broadly in the human transcriptome and emphasizes the necessity of a robust computational filter pipeline to obtain high confidence RNA-DNA difference results when analyzing high throughput sequencing data as suggested in the recent genome-wide RNA editing studies.

## Background

RNA is an important biomolecule that is deeply involved in almost all aspects of molecular biology, such as protein production, gene regulation, and viral replication [1]. In order to perform such a variety of functions, the primary RNA transcripts need to be extensively processed. By changing the genomically encoded sequence at the transcriptional or post-transcriptional level, RNA editing provides an effective way to create transcript and protein diversity with limited primary RNA transcripts in an organism [2-4]. Alteration can occur through the insertion or deletion of nucleotides relative to the original template (insertional/deletional or “indel” RNA editing), or via substitutional RNA editing, in which one nucleotide is replaced by or changed to another.

The most common type of known RNA editing in metazoans involves conversion of adenosine to inosine (A-to-I editing) [5], which is mediated by adenosine deaminase acting on RNA (ADAR) enzymes [6-8]. Inosine preferentially base pairs with cytidine, and is therefore functionally equivalent to guanosine. Thus, A-to-I editing in mRNA can alter the genetic information stored in the primary sequence, leading to changes in protein-coding sequences and mRNA stability and splicing. A large number of A-to-I editing events have been identified in the human transcriptome by genome-wide bioinformatics and high throughput sequencing studies [9-14]. While both coding and non-coding sequences undergo A-to-I editing, it has been found that editing occurs mainly in repetitive sequences which are located within 5’ or 3’ untranslated regions (UTRs) or introns [9-14].

Taking advantage of whole-genome and transcriptome deep-sequencing technologies, recent studies have extensively investigated all the potential types of substitutional RNA editing in the human transcriptome using bioinformatics tools that are capable of identifying mismatches between RNA reads and the matching reference genome [15-17]. While the validity of some of the results in [15] is currently under debate [18-21], these studies revealed a large number of substitutional RNA editing candidate sites including many A-to-I editing events. It is now obvious that combining high throughput sequencing and bioinformatics has the ability to identify RNA editing events that occur at a single nucleotide level across the whole transcriptome.

Insertional and deletional editing events have been discovered in various species [2,4], such as U insertions and deletions in kinetoplastids [22], G and A insertions in paramyxoviruses [23], and various types in *Myxomycota*[24]. Most of these events are found in mRNA sequences, with certain functions like creating new start and stop codons by uridine insertions in kinetoplastids [25], creating new open reading frames by nucleotide insertions in kinetoplastid [26] and *Physarum*[27] mitochondria, and frameshifting between alternative ORFs in paramyxoviruses [23]. In higher organisms, no indel editing events have been identified until recently Zougman *et al.*[28] reported two insertional RNA editing events in human: according to their data in the 5’UTRs of the linker histone H1 mRNA and of the high-mobility group (HMG) mRNA, a single uridine each inserts between an A and a G, creating new translation start sites and producing N-terminally extended proteins. However, to our knowledge no follow-up work has been done concerning these editing sites and no additional indel editing events have been reported in human since.

In this study, we explore the possibility of indel RNA editing events across the transcriptome in human by systematically examining the variations between RNA-seq reads and their matching genome. Specifically, we examine the possibility of genome-wide indel RNA-DNA differences in one human individual, NA12878. We apply different computational tools that use gapped alignments to identify indel differences between RNA reads and the matching genome. While hundreds of such indel candidates are revealed after initial selection, further analysis and filtering indicate that all of them are false positives which result from incorrect alignments including splice junctions, paralog sequences, diploid alleles, and known genomic indel variations. The results from our study suggest that indel RNA editing events are unlikely to exist widely in the human transcriptome and emphasize the importance of thorough filtering in genome-wide studies of RNA editing.

## Results

### Workflow

**Figure 1:**
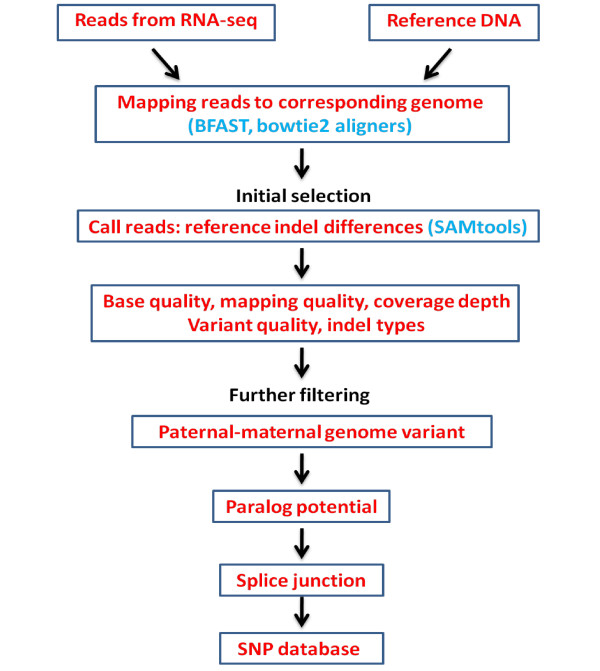
General Workflow to identify reliable indel RNA-DNA differences in the human transcriptome.

Figure 1 shows the general workflow which we follow in our analysis. In short, we first align RNA-Seq reads against their matching genomic sequence. Once suitable RNA-Seq alignments are generated, RNA sequences that are different from the corresponding DNA sequence are identified as initial candidates. These initial candidates are subjected to multiple filters with stringent thresholds to eliminate false positives. The results as well as the considerations taken in developing the approach are described in more detail below.

### Mapping of RNA-seq reads yields hundreds of candidate indel RNA-DNA differences

The initial step in the detection of RNA-DNA differences is the accurate mapping of RNA-seq reads to their matching reference genome. Failure to correctly assign a read to its original location may lead to spurious alignments that may be misinterpreted as editing events. Since a large number of genomic variations including single nucleotide differences and indels exist among different individuals, it is crucial to compare RNA and DNA sequences from the same background (to verify the importance of using the same background we in fact also performed our analysis using the hg19 reference genome and found as expected a large number of genomic variations reported as false positive results). Based on the reference genome (NCBI build 36) and incorporating genomic variations and structural variations identified by the 1000 Genome pilot project [29], the Gerstein lab has recently created a version of the diploid genome sequence for the NA12878 individual from the lymphoblastoid cell line GM12878 [30]. Matching deeply sequenced RNA-Seq data sets for the same cell line are also available. We use this assembled genome to identify possible insertional and deletional RNA-DNA differences in NA12878, by directly aligning RNA-seq reads against their matching genome. Since the assembled genome is a diploid one which contains maternal and paternal haplotypes with small variations in sequences, we first map RNA-seq reads to the maternal genome and list all the potential candidates and then remove the candidates resulting from maternal-paternal genome variations (see below). The detailed information of RNA-seq data and diploid genome for GM12878 we used in this study are described in the Methods section.

The rapid emergence of high-throughput sequencing techniques has resulted in the development of a variety of short sequence read mappers that are based on different alignment strategies. Since our goal is to identify indels within all RNA-DNA differences events, the basic requirement for the mapping tools is that indels should be allowed when aligning short RNA reads to the reference. By evaluating most of the currently available mapping tools, we find that BFAST (Blat-like Fast Accurate Search Tool) [31] is one of the most suitable softwares for our indel analysis. In contrast to some other algorithms that speed up the mapping process by ignoring errors and indels, BFAST is very sensitive to errors, single-nucleotide polymorphisms (SNPs) and especially indels with a considerably fast mapping speed [31]. Since mapping bias inherent to the mapping algorithm may affect results, we also use another tool, bowtie2 [32], a fast and accurate mapping algorithm in which gapped alignment is allowed and compare the results.

For the initial BFAST mapping (alignment settings are described in detail in the Methods section), out of the 113,902,864 reads, 79,833,200 could be mapped to the assembled maternal haploid genome of GM12878 over their entire length. For bowtie2 (using the default setting suggested in the manual) a total of 40,862,987 reads could be mapped to the assembled maternal genome over their entire length. This mapping ratio is significantly lower than that in BFAST, which is probably due to the higher stringency of bowtie2 for mapping a read to the reference genome.

The mapping output for BFAST and bowtie2 are SAM (Sequence Alignment/Map) files, which were processed by the SAMtools software package [33], a package that was originally designed to identify genomic variations. We conduct initial indel variant calling taking advantage of the mpileup algorithm implemented in SAMtools, using the default settings used for calling genomic SNPs except that we do not require “heterozygotes” to reach 50% read support since editing could occur at lower frequency. In order to minimize the influence of sequencing and reverse transcription errors, candidates are required to pass quality control thresholds for base calling quality, read mapping quality (reliability of the alignment across the genome), read coverage, variant/reference quality, and indel type and size (see details in the Methods section).

After these initial selections, 685 candidates remain in the BFAST results while 250 candidates remain in the bowtie2 results. Of these, 110 were shared between the two mapping approaches. The fact that bowtie2 has much fewer candidates than BFAST is probably due to the lower read mapping ratio mentioned above. As for the candidates found by bowtie2 but not by BFAST, many are at the edge of the filtering thresholds. Thus, small differences in the way quality measures and variants of aligned reads are reported in the two mappers lead to a candidate being just above the threshold in one method and just below in the other. We notice, that as indicated below all these questionable reads are filtered out by the additional false positive filtering steps and the overlap between remaining candidates based on BFAST alignments and remaining candidates based on bowtie2 alignments is much larger.

### Careful filtering reveals that all indel editing candidates are false positives

After the initial selections, the list of indel variations called by SAMtools may still contain a large number of false positives that are unrelated to indel RNA editing. These false positives may be a result of known genomic variations, different alleles from diploid genomes, and misalignment of reads due to, e.g., splice junctions and paralog sequences in the genome. We thus apply a series of stringent filters to remove false positives from our candidate lists.

**Figure 2:**
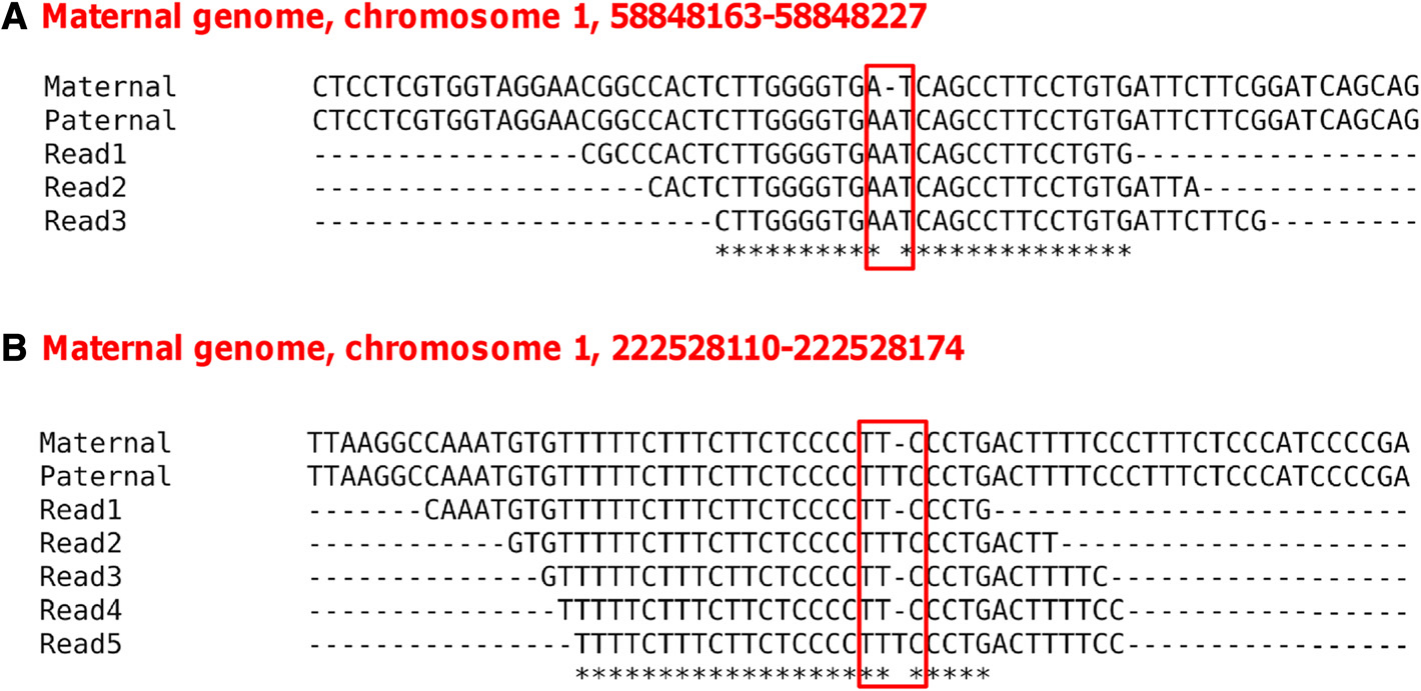
**Examples of false positives resulting from diploid alleles.** (**A**.) All the RNA reads support the paternal genome version; (**B**.) Some of the RNA reads support the maternal genome version while others support the paternal genome version. Note: only part of the RNA reads mapping to the position are shown here.

Since we initially aligned RNA-seq reads to the maternal genome, we first check if the candidate indel RNA-DNA differences result from genuine maternal-paternal genome differences by realigning all the reads that can be mapped to the putative indel sites (including reads that support editing and reads that support the maternal genome version) to the paternal genome using BFAST. This filter identifies 45 sites in the BFAST dataset as well as 60 in the bowtie2 dataset that reflect genuine maternal-paternal genome variations where the apparent indels in the RNA-seq reads reflect the paternal RNA transcript form (see Figure 2 for two examples).

**Figure 3:**
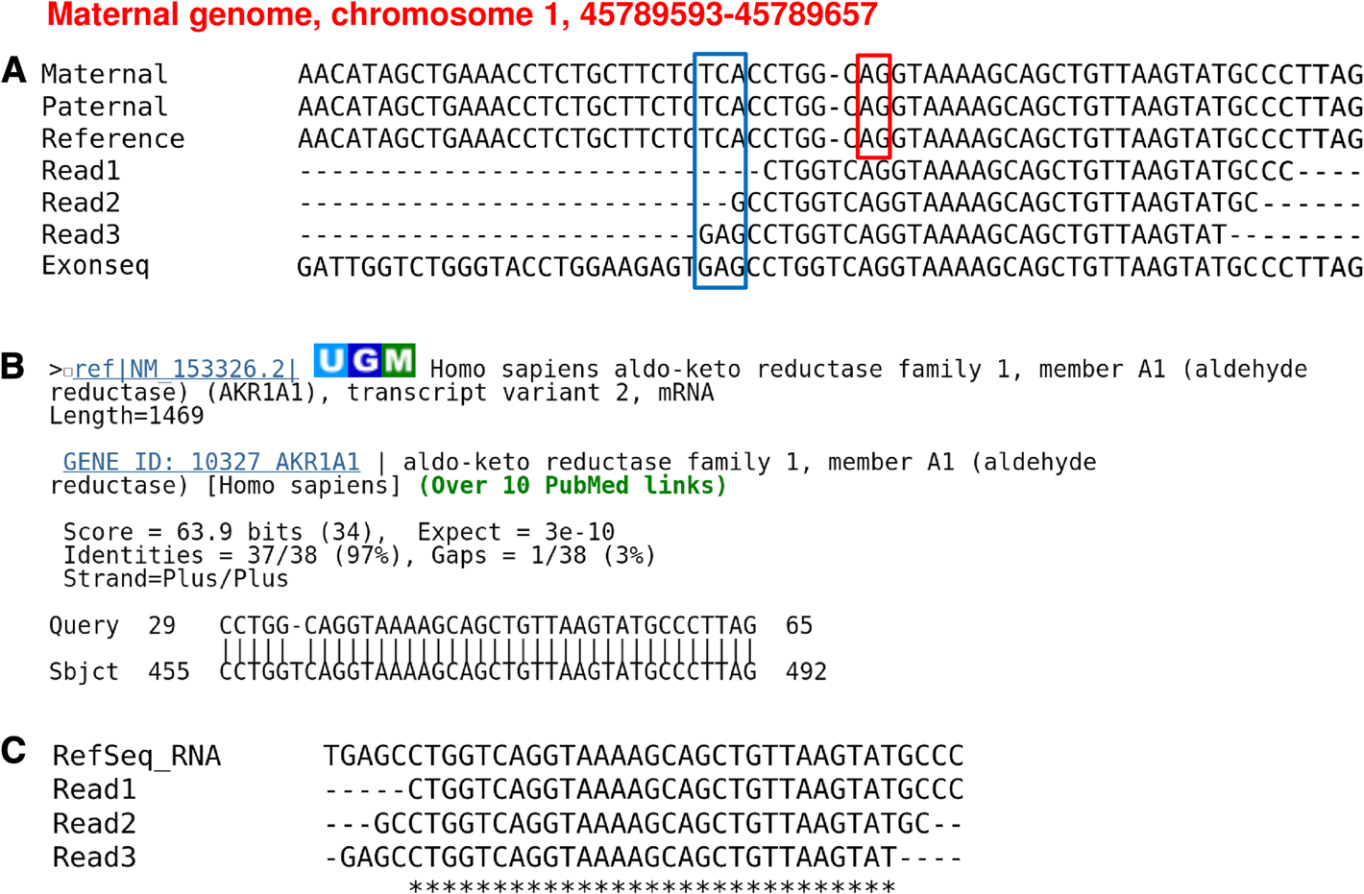
**An example of a false positive resulting from a splice junction.** (**A**.) Multiple alignment of RNA-seq reads and genomic DNA (maternal, paternal genome of NA12878 and NCBI build 37 reference genome) Note: only part of the RNA reads mapping to the position are shown here. The blue box shows the mismatches between RNA-seq reads and the reference genome, but these "mismatches" can be correctly mapped to the exonic sequence. (**B**.) BLAST results of the alignment of the genomic version of the sequence to the refSeq RNA (only part of the sequence can be properly aligned). A further investigation of the mRNA annotations show that the AG in the genomic sequence (marked by a red rectangle in (**A**)) corresponds to a 3’ splice junction. The AG in the reads at that position in the alignment is not the actual 3’ splice signal but an AG in the spliced mRNA that happens to follow the intron and thus can be mapped to the genomic AG splice signal. (**C**.) The reads which support the “indel” can be fully aligned to the same refSeq RNA sequence as in (**B**).

Next, we address possible misalignment due to splice junctions. We use BLAST [34] to search the genomic surrounding sequence (without the indel) from 32bp upstream to 32bp downstream of each remaining indel candidate against the reference genome (NCBI build 37). In addition, we search these surrounding sequences (with and without indels, respectively) against all the currently known splice junctions including the refSeq RNA database at NCBI, Gencode, Ensembl and UCSC genes [17]. We find that a substantial number of initial candidates is due to incorrect mappings of reads across splice junctions, based on the following characteristics: (1) The genome version (surrounding sequence without indels) aligns to the expected position in the reference genome *without indels*; (2) Only *part* of the sequence from the genome version can be properly aligned *without indels* to one of the RNA sequences in the splice junction database (this RNA sequence corresponds to the same position in the reference genome as found in (1), but contains a splice junction close to the putative indel site); (3) The surrounding sequence which *includes the indel* can be *fully aligned* to the same splice junction RNA sequence identified in (2) *without any gaps* (see example shown in Figure 3). Further examinations of these alignments reveal that most of these putative indels are accompanied by one or more mismatches between genomic DNA and RNA-seq reads. Altogether, 609 of the candidate indels in the BFAST dataset are identified as false positives by this filter while in the bowtie2 dataset 169 result from misaligned splice junctions.

**Figure 4:**
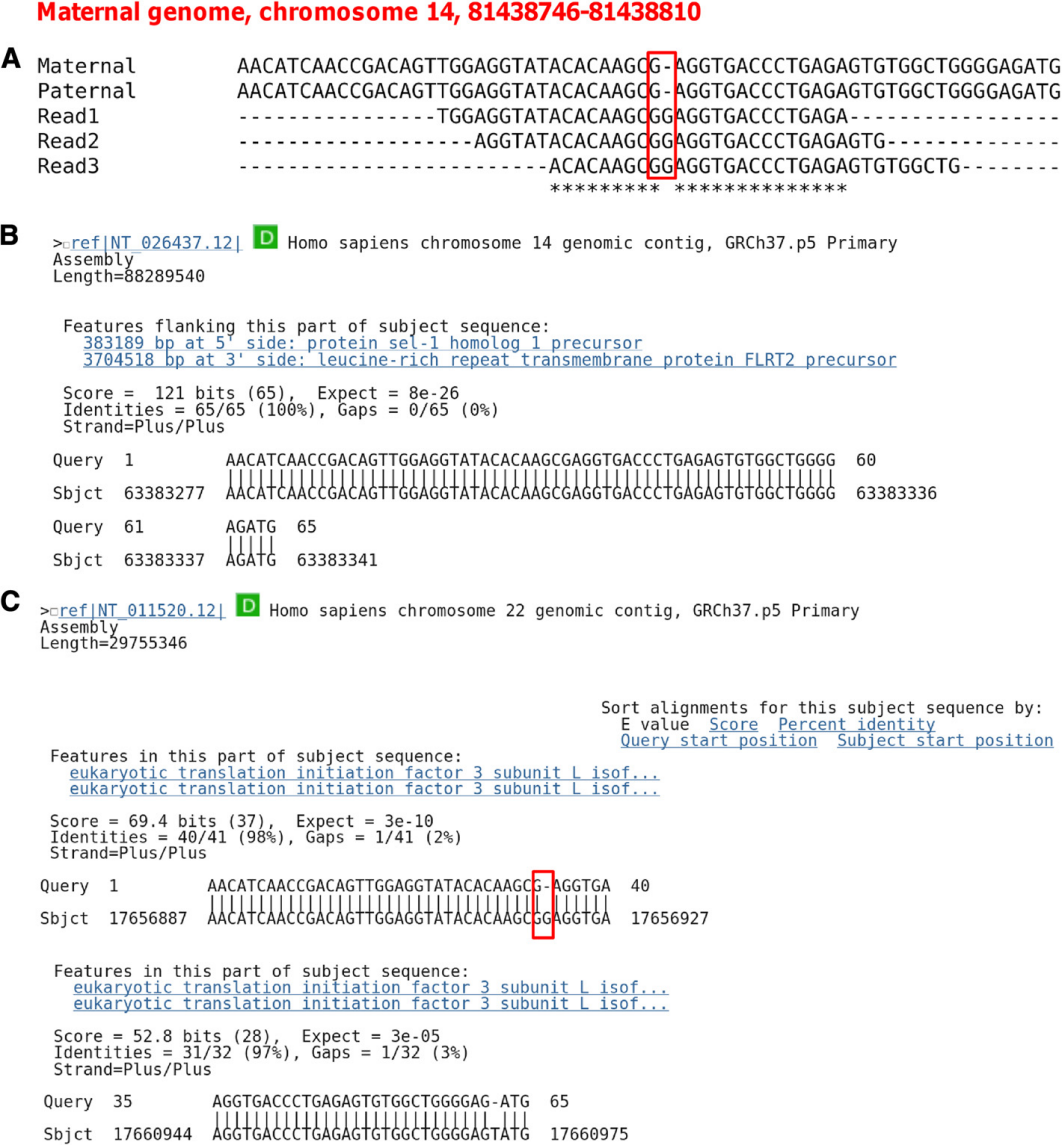
**An example of false positive resulting from a paralog sequence.** (**A**.) Multiple alignment of RNA-seq reads and genomic DNA (maternal and paternal genome) Note: only part of the RNA reads mapping to the position are shown here. (**B**.),(**C**.) BLAST results of the alignment of the genomic version of the sequence to the reference genome (NCBI build 37). It can be aligned to the reference genome at more than one position. The gap highlighted in (**C**) by the red rectangle is the same as the indel difference shown in (**A**).

As suggested in [18], potential paralog sequences may lead to spurious RNA-DNA difference events and thus also need to be ruled out. Therefore, we investigate if some of the candidate indel RNA-DNA differences are signatures of paralog sequences in the genome, by realigning the indel sites and surrounding sequences back to the entire reference genome (NCBI build 37) using BLAST [34]. If an alignment without indels can be found, we declare the candidate as resulting from a misaligned paralog (see example in Figure 4). The reason that we can obtain mappings to different locations when aligning surrounding sequence to the reference genome may be due to the inherent bias in the mapping tools or gaps in the assembly of the NA12878 genome compared to the reference genome. This filter eliminates 19 candidates among those identified by BFAST and 10 candidates among those identified by bowtie2.

**Figure 5:**
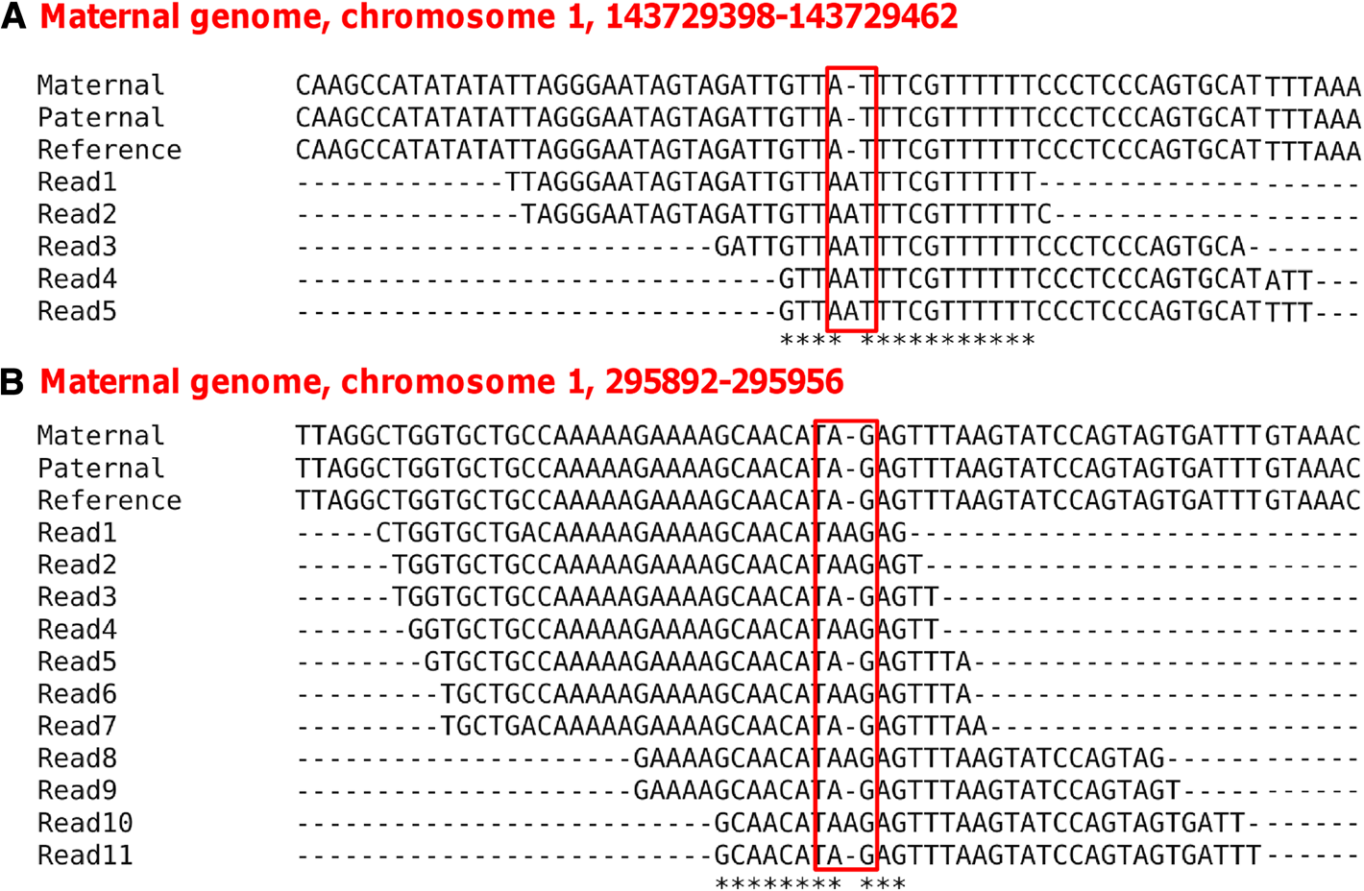
**Alignment of two of the 12 remaining candidates.** These two candidates pass all the other filters but are found in the SNP database (rs56026824 in (**A**) and rs72551074 in (**B**)). The figure shows the multiple alignments of RNA-seq reads and genomic DNA (maternal, paternal genome of NA12878 and NCBI build 37 reference genome) Note: only part of the RNA reads mapping to the position are shown here.

After eliminating false positives from incorrect alignment in the same individual described above, only 12 candidates remain in the BFAST dataset (see Figure 5) and 11 in the bowtie2 dataset within 8 sites common to both. However, these candidates are listed as genomic indel variations in the SNP database. We thus conclude that these most likely represent misassemblies in the maternal and/or paternal genome rather than true indel RNA-DNA differences. Based on this filtering analysis, we conclude that none of the candidates from the initial lists can pass all of the filters.

### Sensitivity of computational pipeline

In order to conclude that our results indeed imply that indel editing is rare rather than being a result of the inability of our pipeline to find indel differences that are present, we tested the sensitivity of our computational pipeline, by examining how many of the known genomic indels in the NA12878 diploid genome can be found by our pipeline before furthering filtering. We first align the NA12878 maternal genome sequences against the paternal genome sequences to locate positions of all the short indels. For all of these sites, we ranked them according to the reads coverage on the site. We found that for the top 100 expressed genomic indel sites (which have read coverages down to 5 reads) 44 sites are found when using the maternal genome for indel calling (where we excluded sites with homopolymer runs of greater than 5 bp which have a higher chance to result from sequencing errors rather than true indel differences). If indel calls from alignments to the maternal and the paternal genome are combined, we found nearly 90% of the covered genomic indels. We thus conclude that the lack of indel editing sites found in our study is not due to a lack in sensitivity of the pipeline.

### Additional RNA-seq datasets yield consistent results

In order to study the dependence of these results on technical details such as read length, we examine other RNA-seq data sets from a recent study [30] on the same cell line GM12878 (for a detailed data description see the Methods section) in addition to the RNA-seq data described above. We perform the same analysis as described above for the 54bp single-end short reads in this data set but limit ourselves to BFAST only since this produced more candidates on the first data set. The results are summarized in Table 1. We find that 1037 sites pass the initial selection (197 of which overlap with the candidate lists from the first data set). Applying the different filters again identifies all of them as belonging to one of the four categories: genuine paternal allele, mismapped splice junction, mismapped paralog sequence, and genomic variation annotated in the SNP database.

**Table 1:** Summary for indel candidates analysis of additional RNA-seq datasets

	
Initial candidates	1037
Paternal alleles	95
Splice junctions	907
Paralog sequences	10
Known genomic variations	25

## Discussion

In this work, we provide the first systematic study of the possibility of genome-wide indel RNA-DNA differences in one human individual, NA12878, whose RNA and matching genome have been deeply sequenced. We applied different computational tools that are capable of identifying indel differences between RNA reads and the matching reference genome. After initial selection using SAMtools, we found hundreds of such indel candidates. However, with careful further analysis and filtering, we found that all of them are false-positive results such as splice junctions, paralog sequences, different alleles from diploid genomes, and known genomic indel variations from the SNP database. We thus conclude that there is no evidence for widespread insertional or deletional RNA editing in the human genome.

However, it should be noticed that the RNA-seq data sets we used are from a particular lymphoblastoid cell line; it is thus in principle still possible that widespread indel RNA editing events could be cell type specific and that we may have missed them by selectively focusing on the lymphoblastoid cell line. Moreover, our stringent requirement for detecting such events (at least 2 RNA-seq reads with high base quality and mapping quality supporting editing) may have missed potential sites which are edited at very low frequency.

It is interesting to relate our findings to the recent discussions on substitutional RNA editing initiated by Li et al. [15]. Several technical comments on that study [19-21] pointed out that the mismatches of RNA-seq reads to the reference genome are almost exclusively at the ends of sequencing reads. The response by Li et al. [35] proposes that one of the reasons resulting in this bias is co-occurrence of substitutional RNA-DNA difference sites with insertion/deletion RNA-DNA differences sites. Our results here indicate that such widespread indel RNA-DNA differences are unlikely to exist. Rather, our finding of false positives resulting from splice junctions that often combine apparent mismatches and indels seems to provide a possible explanation for the coexistence of mismatches and indels as well as their occurrence at the end of the reads. Thus, our observation further questions the proposal of indel RNA-DNA mismatches in [35] to explain the end effect of mismatches.

The absence of indel RNA editing in our study also has to be discussed in the light of the previous study suggesting two potential insertional RNA editing sites in human [28]. This apparent discrepancy led us to specifically revisit the two insertional RNA editing sites identified in [28]. Their work suggested that, a single uridine each inserts between A and G in the 5’UTRs of linker histone H1 and high-mobility group (HMG) mRNA and creates new translation start sites and produces N-terminally extended proteins. Further examination of their study and our analysis allow us to propose several possible reasons for this discrepancy.

First, as mentioned above, the editing events may not occur in the specific cell line we investigated. Moreover, the study in [28] showed that in certain cell types the abundance of the “edited” form of proteins is much lower than the normal form of proteins; thus, it is possible that the coverage of RNA-seq data we used is not enough to detect the editing events which occur at a low frequency based on our filtering criteria. In fact, our alignment and filtering data show that only one RNA-seq read can be reliably aligned to the “AG” position in the 5’ UTR of H1.0 mRNA without insertion for both, BFAST and bowtie2, results. We note, that this is not due to the lack of a polyA tail on the histone H1.0 mRNA when preparing the sequencing libraries, since the synthesis of histone H1.0 is not cell cycle-regulated and its mRNA is polyadenylated [36,37]. For the other case, HMGN1, around 10 reads can be reliably mapped and none of them contain the insertion site. This indicates that the read coverage at these two sites may be not sufficient to identify the “edited” version of the RNA (according to [28], for *h1.0*, 11 of 301 EST sequences support the “edited” version; while for *hmgn1*, only one EST sequence supports the “edited” version).

**Figure 6:**
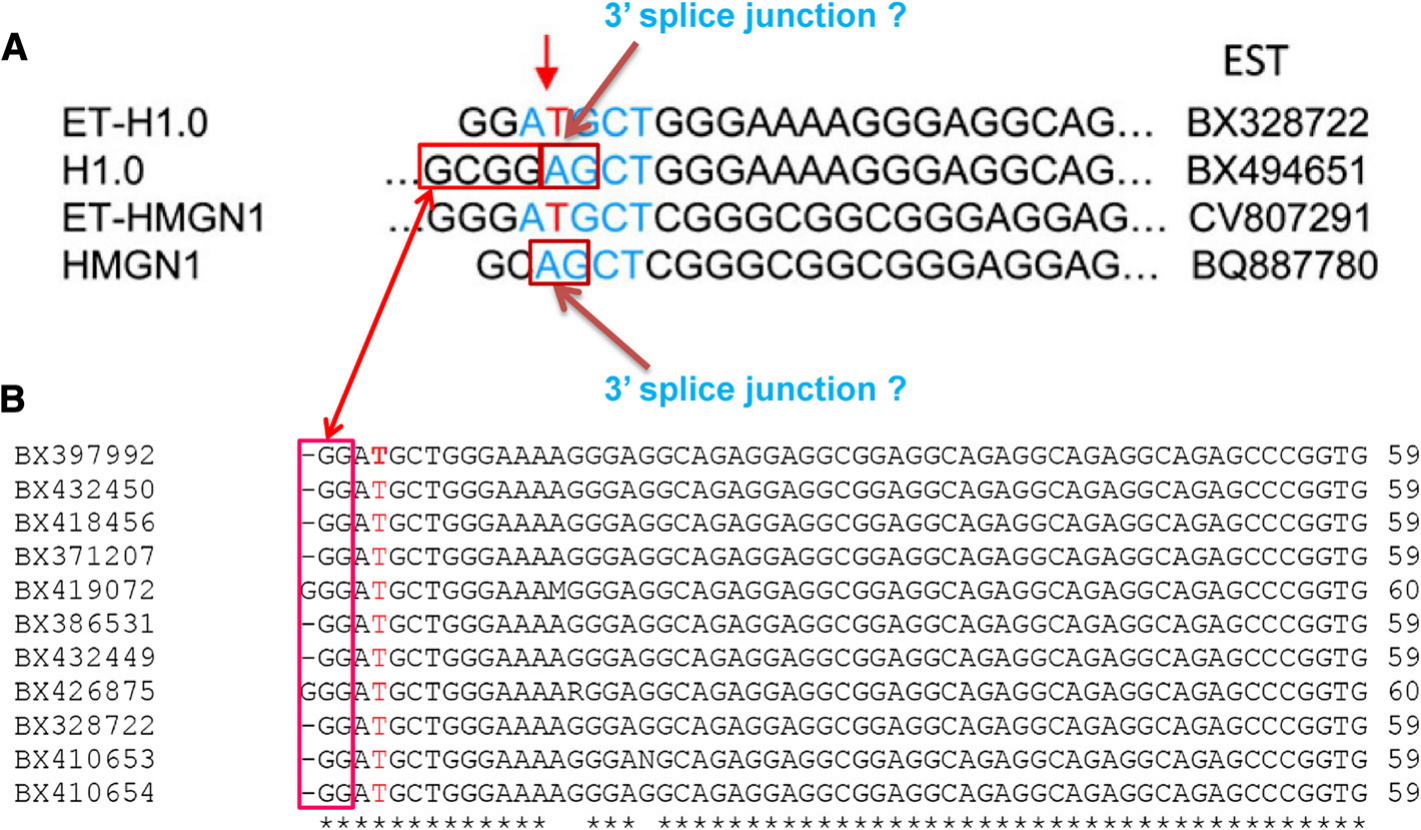
**Proposed explanation for previously identified insertional RNA editing sites in human.** Based on our indel analysis of false positives and EST data in [28], we hypothesize that the two “insertional RNA editing sites” may be signatures of novel splice junctions. For *h1.0*, the base "G" in the EST sequence is a mismatch compared to the genomic base "C" which is present in all EST sequences which have 4 bp upstream of the "editing site". For *HMGN1*, the only one EST sequence which supports "editing" also displays a mismatch ("G" in the genome and "C" in the EST sequence) upstream of the "editing site". The "AG" marker in the red box in the *H1.0* and *HMGN1* sequence may serve as an unknown 3' splice site.

However, the results of our filtering analysis promote us to also consider the possibility that these apparent “insertional editing sites” could be signatures of other biological “artifacts”, such as a paralog sequence or splice junction. We thus examined if these sites could result from paralog sequences in the genome but found no such paralog sequence. However, when examining the EST data which were used as evidence for editing sites ( [28] found 301 ESTs carrying the H1.0 5’UTR, 11 of which contain the U insertion, and only one EST sequence supporting a U insertion in HMGN1), one unusual property is observed: All of the 11 sequences for H1.0 have a very short 5’ surrounding sequence (exclusively 3~4bp) at the “editing site” (see Figure 6). For two of the 11 sequences which have 4 bp upstream of the editing site, the first base is “G” which does not match to genomic version “C”. For HMGN1, the only supported sequence contains several mismatches at the upstream surrounding sequence between the genomic sequence and the EST sequence.

This is very similar to the pattern observed in our “splice junction” false positives in which “indels” occur close to the end of the alignment and coexist with mismatches. This observation thus may imply that it may have resulted from rare and so far unknown splicing events. Again, we note that histone 1.0 belongs to replication-independent histone mRNAs [36,37] and thus could in principle be spliced even though no such splice variant has been documented so far. Moreover, the original study [28] indicated that the “edited” form of H1.0 protein colocalizes with splicing speckles which may suggest a connection to splicing. Since [28] did not directly sequence the DNA and corresponding RNA surrounding the “editing sites”, careful examination revealed that splicing can also explain all the additional experimental observations in their study, i.e., an extended protein form, restriction enzyme digestion, etc. Therefore, it is possible that these only two “insertional RNA editing sites” so far are indeed results of novel splicing events, which would require experimental verification.

## Conclusion

In this study, we systematically examined the possibility of genome-wide indel RNA-DNA differences in one human individual, NA12878, by aligning several RNA-seq datasets to the corresponding assembled diploid genome from the same cell line. The initial selection revealed a number of indel candidates; however, following analysis showed that all of them are unrelated to RNA editing. Overall, our study suggests that the previously proposed insertional RNA editing events are unlikely to exist in the human transcriptome and that to obtain high confidence RNA-DNA difference results, it is necessary to build a robust computational filter pipeline when analyzing high throughput sequencing data.

## Methods

### Reference genome and RNA-seq reads (Data sources)

The assembled diploid genome sequences of the individual NA12878, genome annotations and corresponding variants information (between the maternal and paternal sequences and the reference genome NCBI36/hg18) were downloaded from http://sv.gersteinlab.org/NA12878_diploid/. Illumina generated RNA-seq data from the same cell line (lymphoblastoid cell line GM12878) as the one used for assembly of the diploid genome sequences of the individual NA12878 were downloaded from the NCBI Sequence Read Archive (SRA). In order to verify the robustness of our results with respect to sequencing parameters such as read length, other single end RNA-seq datasets were downloaded from NCBI GEO (GEO accession number GSE30401). The latter RNA-Seq data sets were generated as part of the ENCODE Project [38]. Table 2 provides the exact identifiers of the data sets.

**Table 2:** First round RNA-seq data used in this study

**SRA accession number**	**Run used in this study**
SRX000565 (33bp)	SRR002055, SRR002063, SRR005091, SRR005096
SRX000566 (33bp)	SRR002052, SRR002054, SRR002060
**Second round RNA-seq Data used in this study**	
SRX082145	SRR306998, SRR306999, SRR307000, SRR307001, SRR307002, SRR307003, SRR307004

### Mapping RNA-seq reads to the corresponding reference genome

RNA-seq reads were first aligned to the maternal-derived haploid genome sequences using the standard pipeline of the Blat-like Fast Accurate Search Tool (BFAST) [31]. As one of the computational tools that is capable of discovering indels with gapped local alignment, BFAST is very sensitive to errors, SNPs and especially indels with a considerable mapping speed [31]. Specifically, ten indexes recommended for aligning reads with length less than 40bp in the BFAST manual (see Table 3) were used to index the reference maternal haploid genome.

**Table 3:** Index sets in BFAST used in this study

**Index number**	**Mask**
1	111111111111111111
2	111010001110001110100011011111
3	11110100110111101010101111
4	11111111111111001111
5	1111011101100101001111111
6	11110111000101010000010101110111
7	1011001101011110100110010010111
8	1110110010100001000101100111001111
9	1111011111111111111
10	11011111100010110111101101

Most of the parameters in the alignment process were set to their default values. A single lookup is ignored if it returns more than K=8 candidate alignment locations (CALs); the maximum number of CALs for a read was M=1280. Local alignments were performed for each CAL using default settings and nucleotide substitutions, insertions and deletions were identified in the gapped alignment. Alignments were prioritized by alignment score and only the highest scoring alignment for each mapping read was output. The mapping output was set to SAM format.

### Post-processing of mapping output and variant calling

To identify RNA editing sites, the output RNA-seq alignment files in SAM format were processed by the publicly available, open source SAMTools software package [33] ( http://samtools.sourceforge.net/) for variant calling. The version we used in this study was samtools.0.1.17.

Using SAMTools, the output SAM files were first converted to their binary versions (BAM files) and then these BAM files were sorted and indexed for rapid lookup. The sorted BAM files were further processed in the variant calling step: using the “mpileup” function in SAMTools, indexed reference sequences and position sorted bam alignment files generated files with read information at sites where mismatches and indels from the reference sequence were detected. Then, only information for indel differences was kept, while reads that contained only mismatches were discarded. The output file after this step served as the starting dataset for the indel RNA editing analysis.

### Initial filtering of indel variants

To eliminate false positive results due to sequencing and reverse transcription errors, the initially identified indel variants were further filtered by the following criteria:

1. Base quality filter: remove bases at the indel site with a sequencing quality score below 20.

2. Mapping quality filter: remove reads with a mapping quality score below 20; discard a read if the indel position is within 2bp of the 5' end or 3' end; discard an indel-containing read if more than 3 mismatches are present.

3. Coverage depth filter: remove candidates with less than 2 indel-containing nonduplicated reads; remove candidates with less than 5 reads; remove candidates with less than 5% indel-containing reads of the total covering reads.

4. Variant quality: remove candidates with QUAL Phred-score of variant calling below 0.01.

5. Indel type and size filter: remove variant sites that display more than one nonreference alleles as well as variant sites that contain any uncertain bases (“N”); only keep candidates with only one nucleotide difference from the genomic DNA (i.e., indel size should be one); remove variant sites that display homopolymer runs of more than 5 identical nucleotides.

## Abbreviations

BLAST: Basic local aignment search tool; BFAST: Blat-like fast accurate search tool; NCBI: National center for biotechnology information; RNA-seq: RNA sequencing; SAM: Sequence alignment/map; SNP: Single-nucleotide polymorphism; SRA: Sequence read archive.

## Competing interests

The authors declare that they have no competing interests.

## Authors’ contributions

CC was involved in the conception of the study, performed all the computational work, and drafted most of the manuscript. RB conceived and directed the study and revised the manuscript into its final form. All authors read and approved the final manuscript.
